# A Treatise on Puerperal Fever, Illustrated by Cases Which Occurred in Leeds and Its Vicinity, in the Years 1809-12

**Published:** 1816-05

**Authors:** 


### PART II.

### COMPREHENSIVE ANALYTICAL REVIEW
OF
### MEDICAL LITERATURE.

"Tros, tyriusve, *nobis* nullo discrimine agetur."

*A Treatise on Puerperal Fever, illustrated by Cases which occurred in Leeds and its Vicinity, in the Years* 1809-12, By WILLIAM HEY, Jun. &c. &c. Octavo, pp. 238. 1815.

THE discrepancies of opinion respecting the nature and treatment of Puerperal fever, which have prevailed in all ages among medical writers and practitioners, must tend to embarrass the younger classes of the profession in their first outset, and indeed for a long period afterwards; since the fatality of the disease will cause much vaccillation in the treatment of it. We have therefore much pleasure in portraying the contents of a volume from so very respectable an authority as that of Mr. Hey, in which a practice is recommended that is easy of application and successful in its result. Mr. Hey candidly confesses himself indebted to Dr. Gordon's excellent treatise on the same subject, for the plan of treatment he adopted; and

"Though he has little that is new to add to this plan, yet since the practice in puerperal fever is far from being settled, whatever may throw additional light on the complaint, or on any treatment that has been adopted for its cure, cannot be thought superfluous.

Mr. Hey alludes to the *coincidence* of curative views entertained by Dr. Armstrong and himself, as a corroboration of their justness.

Our author defines Febris puerperalis, in its most simple state, to be "fever in child-bed, accompanied with pain which has no complete intermission, and extreme soreness in the abdomen." These are the invariable, or pathognomonic accompaniments; but there are various other symptoms more or less commonly joined with them. While some authors have looked upon this disease as purely inflammatory, others have considered it typhoid, and others still as mixed. Its causes have been still more variously accounted for. Obstructed lochia — translated milk — injury in labour — cold — too early exertion — foulness in the primæ viæ — absorption of putrid lochia — foul air — contagion, &c. &c. have all been accused in their turns. Mr. Hey wishes to avoid all theoretical speculations, and "to write entirely as a practical man." He has found the nice distinctions of authors unavailing, sometimes prejudicial at the bed side.

"An attention to them, says he, had no inconsiderable share in restraining me from adopting that method of treatment which afterwards proved so eminently successful."

He considers, perhaps justly, that all the varieties described in books, are only different grades of the same disease, and only requiring a more or less decisive employment of the same remedial means. When the disease is epidemic, it is of course more fatal and intractable than when sporadic, and must be met with more energy in the attempts to cure. In the following history and cases, the result of his own practice is stated, with occasional remarks on the observations of other writers.

History. The epidemic here described, commenced at Leeds, in the month of November 1809, continuing with different degrees of severity, and some intermissions, till Christmas 1812. During this time, it was not confined to the town and immediate vicinity, but spread to the country and neighbouring towns. In these, it proved as fatal as in Leeds. It was not peculiar to any particular constitution or temperament, but promiscuously seized on all. The strong and the weak, the robust and the delicate, the old and the young, the married and the single; those who had easy and those who had difficult labours, were all equally, and indiscriminately, affected. In the begining, it chiefly affected women in the higher situations of life; and in its progress they were equally liable to its attack as the lower orders. It was not affected by the sensible qualities of the air, since it prevailed equally in cold and hot weather; in wet and in dry seasons. From this, it is not inferred that the epidemic is unconnected with some peculiarity in the constitution of the atmosphere for the time being. No contemporaneous epidemic prevailed with puerperal fever, excepting erysipelatous inflammations; many cases of which, were of a very malignant kind. The same was remarked in the epidemic at Aberdeen some time before, by Dr. Gordon. Mr. Hey did not meet with a single instance, where this disease succeeded a preternatural delivery; or even a particularly hard labour. On the contrary, it most frequently occurred "after the most easy and natural labours."

Symptoms. The period of attack was *usually* 48 hours *post partum*; till which, the patient was perfectly well. Then came a rigor, succeeded by great heat; often terminating in profuse perspiration, and severe pain in the abdomen. The pain had no complete intermission; sometimes no remission: but it commonly had exacerbations resembling the throes of labour. In the remissions, pressure on the abdomen occasioned great uneasiness.

The pulse early became rapid; sometimes strong and full; more frequently weak, or soon losing its strength. Very soon after the attack, it ranged from 110 to 150 pulsations in the minute.

Headache, giddiness, sense of confusion, ringing of the ears, very often took place. Pallor of the countenance and expression of anxiety succeeded the hot stage. The febrile heat was very various; sometimes the skin was quite cool and pale; generally hot and dry: sometimes perspirable. Tongue most commonly unaffected: sometimes white and furred.

If the disease commenced *prior* to the secretion of milk the *latter* was entirely prevented. If *posterior*, the milk disappeared, and the mammæ became flaccid. The lochia were variously affected; sometimes unaffected; consequently affording no criterion.

In bad cases, a vomiting or nausea came on early; but in general, it appeared late in the disease. The bowels were easily opened at first; but if the disease had continued some hours, or gained ground, they were with difficulty moved. Towards the end of the disease, a dysenteric purging was common, without blood. The pains were frequently succeeded by a motion.

"A degree of fulness in the hypogastric region was often evident from the first attack; and not unfrequently the uterus could easily be perceived forming a distinct tumor above the pubes."

Pressure here gave great pain. In six or eight hours, if the patient was not relieved, the swelling began to extend itself to the whole of the abdomen; when the uterus was lost in the general tumefaction. A diminution of size in the uterus was favourable.

Dyspnœa, and decubitus difficilis, excepting on the back, were the consequences of the abdominal distension. A mitigation of these augured favourably.

When the disorder was not checked, great prostration quickly supervened. The pulse was innumerable; the tongue sometimes brown; the cheeks were flushed; the countenance wild, and expressive of great distress; the body covered with a clammy sweat. Now, the violent abdominal pain often ceased; but the distension occasioned pains in the back, sides, chest, &c. The patient became restless, while vomiting, hiccup, delirium, &c. were the harbingers of dissolution, which usually closed the scene in a few days from the commencement of the attack.

This was the *usual* course of the disease; but the aberrations were numerous, and occasioned much embarrassment. These varieties we shall record.

In the period of attack. This varied from twenty hours, to six days, *post partum.* In a large majority, however, the period was about, or within 48 hours. Two cases of the epidemic were met with after abortion; one at three, the other at six months. In general, the danger was great in proportion to the earliness of the attack; but there were many exceptions.

In the rigor. Though rigor was commonly the first symptom, yet some of the worst cases were unattended throughout with any shivering. Nevertheless, those which began with rigors, were most alarming and rapid in their progress; but the symptom itself so early pointed out the nature of the disease, that no time was lost; and consequently the severity of the attack was more thancounterbalanced by this knowledge. When the pain came on without rigor, it was more frequently taken for after-pain, and thus, some precious time was lost. In some cases, the rigor returned several times; even where the case terminated favourably.

Pain. "This was a very deceitful symptom; and when it was not preceded by rigor, occasioned great embarrasment by the irregular manner of its attack; and the consequent difficulty of distinguishing it from after-pains. When the disease commenced with a shivering fit, violent pain and extreme soreness of the abdomen generally accompanied, or immediately succeeded the shivering. But sometimes, pain was the first symptom; and then it would often come on by paroxysms, and appear equivocal in its nature; having such long intervals as to induce the hope, that it might not return; or that it might arise from some other cause than inflammation. I have several times known an interval of six or eight hours, or even more, after the first attack of pain." p. 30.

What tended also to embarrassment was, that during the epidemic, after-pains were more than usually violent; and in some few cases, were not easily distinguishable from slight attacks of puerperal fever. Again, when the pain of puerperal fever did come on in this irregular way, the pulse was not immediately affected; though it afterwards became as rapid as when the disease had come on with rigor.

"The seat of the pain, in the beginning, was most commonly in the hypogastric region, just above the pubes; sometimes in one side, and in the right and left indiscriminately. It frequently shot into the back, hips, and thighs, and even to the extremities of the toes. The principal seat of pain was occasionally in the groin, where the round ligament of the womb emerges." 31.

Affection of the head. The intellectual faculties were seldom impaired. The head was sometimes free from all complaint; but generally affected with pain, giddiness, or sense of confusion: the latter, occasionally, though rarely, producing slight alienation of mind. Real delirium never occurred till near the approach of death; and often not then.

The tongue was never incrusted with the dry brown fur of typhus, except the disease was protracted, or improperly treated. It was generally moist and soft; often but little altered from its natural appearance, even in bad cases.

The blood was almost invariably sizy; and the crassamentum firm.

*The termination of the disease,* whether favourable or not, was very uncertain in its period. If early seen and vigorously treated, it was either arrested at once, or brought to a crisis within twenty-four hours. Even if a delay of twelve hours occurred, provided the subsequent energy of treatment was proportionally augmented, the fever was generally cured in two or three days. If neglected, or inadequately treated in the beginning, the disease was protracted to five, and from that to fourteen days, but seldom so long as a week. "When the termination was unfavourable, it was sometimes delayed to eight or ten days; and in one case, till six weeks after the attack. It generally, however, proved fatal in two or three days, sometimes in twenty-four hours."

Method of cure. In every case of accouchement, it was Mr. Hey's practice to give a purgative (pulv. jalap. gr. xxv. submur. hydrarg. gr. iij) on the day succeeding delivery. When the disease has actually commenced,

"The method of cure consisted in large evacuations by bleeding and purging; and although other remedies may sometimes be useful auxiliaries, *these* are indispensable; and they alone will generally be found sufficient, if they are employed in a proper and seasonable manner."

The following abstract from Dr. Gordon's Treatise will convey a good idea of both gentlemens' practice.

"The method which I found most successful was by copious bleeding soon after the attack of the disease. But this did not answer the end unless it was performed early, and in large quantity. When I took away only ten or twelve ounces of blood from my patient, she always died; but when I had courage to take away twenty or twenty-four ounces at one bleeding, in the begining of the disease, (i.e. within six or eight hours of the attack) she never failed to recover. After bleeding, it was my practice to give some active purgative, on purpose to bring on a diarrhœa; which, when excited, I found necessary to continue through the whole course of the disease, till it was entirely conquered. And it is a matter of the utmost moment, to prescribe such purgatives as will operate with all possible speed. I found that most dependence was to be put in calomel and jalap; three grains of the former, and two scruples of the latter, were mixed with conserve of roses, and made into a bolus, which I always administered immediately after bleeding. After this, a purging mixture was given in such proportions, as to produce five or six motions every day, without intermission, for the first three days of the disease; after which, I diminished the dose, but still continued the medicine, till the disease totally ceased. Every night I administered an opiate, in order to give a respite to nature, and strength to the patient, to enable her to bear the evacuations, which she must necessarily undergo the ensuing day. In this manner I treated my patients; and the same method, if followed by others, will, I am confident, be attended with equal success. With respect to the prevention of the disease, he says, "The purging bolus which was so effectual in the cure, was equally efficacious as a preventative. This bolus was given the day after delivery, in the morning; and all who got it, except one, escaped." Gordon on Puerperal Fever. p. 77, 80, 85.

When Mr. Hey was called at an early period, he seldom took away less than twenty-four ounces of blood at first; unless delicacy of constitution, or previous evacuations forbade it. If a delay of eight or ten hours had occurred, or the symptoms were unusually severe, a quantity to the extent of thirty, forty, or even fifty ounces was taken. When the abdominal pain and soreness were not removed, or materially alleviated in six hours, the bleeding was repeated; nor was this prevented even by faintness or deliquium. In short, there was no safe limit to venæsection but the removal or relief of pain, within the first twelve hours.

Immediately after blood-letting, cathartics are to be employed. Mr. H. generally gave ʒß of jalap, with three or four grains of calomel in a bolus; followed up by a cathartic solution taken every hour or two till copious evacuations followed. The solution was continued afterwards, so as to keep up a moderate carthàrsis for two or three days if necessary; allowing it gradually to subside.

Dr. Gordon gave opiates at night; Mr. Hey never found advantage from them, as they afforded but an insidious truce.

"It became my practice, therefore, to continue the purging literally *without intermission*, and without the interposition of opiates; and to this, in conjunction with more copious bleeding, I attribute the more speedy termination of the disease. These means, when early employed, were generally sufficient; but circumstances occasionally occurred to make other auxiliaries useful. These were emollient glysters; fomentations of warm water. Saline draughts, in a state of effervescence, are refreshing to the patient between the purgatives. If these fail, grateful cordials may be given in the latter stages of the disease, to alleviate the distressing feelings of the patient: but cordials or tonics can afford no other advantage. The mischief which has taken place in the cavity of the abdomen, whether by extravasation, suppuration, or gangrene, renders the disease incurable."

Some rare exceptions to this are related by Dr. Gordon; one, where the confined fluid made its way by a direct outlet; two, where it escaped at the umbilicus; and one, by the urethra. Purging may be carried further, and employed at a later period than blood-letting. Even after serous effusion, purgatives offer some prospect of success.

We now come to the cases illustrative of the foregoing observations. They certainly are not written with Hippocratic terseness, and therefore we shall be obliged, in conformity with the principle by which we are actuated, to *abridge* some of them, in order to make the analytical picture of the work before us as complete as possible.

Case I. A young married lady, delivered safely of her first child on the evening of December the 9th, 1809, remained well till the afternoon of the 11th, when rigor occurred. When visited, skin perspirable; pulse 120, not full; little pain or soreness of hypogastrium; "cooling purgatives, salines, and opiates, as the peculiar circumstances seemed to require." This treatment seemed at first successful; but in a few days the pains returned with great violence; with abdominal tumefaction; obstinate diarrhœa, &c. And the patient died on the tenth day.

Case IV. A robust woman was delivered of her first child at four o'clock on Sunday afternoon. Monday, a saline purgative, by way of prevention. Tuesday, at two in the morning, seized with the disease. The pain was very severe, with a full, strong pulse. A purging draught with sulph. sodæ and manna in infus. sennæ—A carthartic enema. In a consultation, eight leeches were ordered to the abdomen. At 3 P. M. the pulse began to lose its strength—Distension of the abdomen advanced rapidly; obstinate vomiting supervened, and the disease finished its fatal career in thirty-five hours from the commencement.

CASE V. Was one of great rapidity. A young lady, delicate, but enjoying good health, was delivered, after an easy labour, at three, P. M. Jan. 26, 1810. At nine, same morning, pulse 72. Slight after pains, which soon subsided. Passed a comfortable night. At five in the morning awoke from sleep with pain in the region of the uterus, accompanied with a chill, and succeeded by heat. Pain came on in paroxysms, and shot into the hips, but without complete intermission. At nine, A. M. some fulness in the abdomen, with exquisite sensibility in the hypogastrium on pressure. Pulse 120; tongue clean. A purging draught with sulph. mag. et mannæ aa ℥ß. A purgative enema. Part of the draught only was taken; the other rejected. At one, P. M. the pulse 130; at three P. M. abdominal distension and frequency of pulse increased; the pain not so violent as in some other cases. A warm fomentation procured a temporary relief; purging draught operated well. ℞. Decoct. cinchonæ ℥x. ammon. carb. gr. viij. M. ft. haustus 2nda quaque hora sumendus cum coch. puerile julepi infra præscripti.

℞. Succ. limon. recent. ℥ij. Spt. lavend. comp. gtt. xxx. M. At eight, P. M. she appeared dying; and at ten the same evening expired, seventeen hours from the commencement of the disease, and forty-three hours after delivery.

CASE VIII. Shows a strong disposition to puerperal fever before delivery.

CASE XII. Brings us to a view of the new method of treating this formidable epidemic; and we shall condense it for our Readers.—Mrs. W. a delicate young woman, was delivered of her second child at nine, P. M. of the 8th of August, 1810. From imperfect contraction of the uterus, a considerable quantity of blood was lost. 10th, in the morning, took xxv grains of jalap and three of calomel, which procured ten stools. 11th, at two in the morning, seized with puerperal fever. Violent rigor, immediately succeeded by severe and continued pain in the abdomen. At six, A. M. body swelled, and excessively tender; violent head ach, and throbbing of the temples; pulse 140. Venæsecto e orificio magno ad ℥xvj. which occasioned faintness. The pain abated during the sanguifluxus, and became periodical with complete remissions, gradually decreasing in severity. At eight, A. M. half a drachm of jalap in a bolus. At nine, the pains began again to increase. A consultation caused delay. At ten, the orifice again opened, and four ounces of blood abstracted. The former blood exhibited the inflammatory crust, with firm crassamentum. An enema exhibited; a large blister to the abdomen; saline draughts with sulph. mag. et liq. ant. tart. every two hours.

After the second bleeding, the pain gradually abated; the patient fell asleep at one, P. M. and awoke at two, quite easy; from that time the pain returned no more. Purgatives completed the cure.

The following case will so clearly demonstrate the safety and the utility of blood-letting in puerperal fever, that we shall transcribe it entirely; as it will render the insertion of any others unnecessary.

CASE XX. "June 4th, 1812, about noon, Mrs. ——— who was young and of a good constitution, was delivered of her first child, after a natural labour of sixteen hours. On the following day the purging bolus was prescribed; but I have since learnt that is was not taken. She had scarcely any afterpain, and the secretion of milk took place at the usual period; but was rather scanty. She remained well till the 9th, at noon, five complete days; when she was seized with a severe pain in the abdomen, resembling labour-pain. We were immediately sent for; but, my father and I being absent from home, one of our pupils saw her The pain having abated, and the pulse being no more than 86 in a minute, he only requested that we might be informed if the pain should return. No message was received; but my father visited her at four, P. M. The pain had returned a short time before, and was so acute, as to cause her to cry out. The abdomen was exquisitely sensible between the paroxysms of pain; and the pulse had risen in the space of four hours to 134 (forty-eight in a minute), though the pain had been absent nearly the whole of the time. The head was not affected either with pain or confusion. He immediately took away from the arm thirty-ounces of blood. As the bowels were in an open state, two evacuations having taken place in the morning, in consequence of a laxative prescribed on the preceding day, instead of the usual purging bolus, he ordered the cathartic solution, with a grain of antim. tart. in eight ounces, of which three table spoonfuls were to be taken every two hours. Ten P. M. The pains had continued at intervals, till seven o'clock;

Since that time they had nearly ceased; but the soreness of the abdomen had not abated, although three loose stools had been procured; and as the pulse was full, and beat at the rate of 120, it was thought advisable to take away ten ounces more of blood. The pain returned soon after the bleeding, though in a much slighter degree than at first. The patient also complained much of sickness, which I attributed more to the antimonial than to the loss of blood. Between twelve and one o'clock she had two more stools, and the sickness increasing, the contents of the stomach were also evacuated. For a while after the vomiting, she was comfortable and easy; but the pain having returned, I had proposed at two o'clock to open the vein a third time; however, during the necessary preparations, the pain ceased and she fell asleep.

I sat by her an hour, and then, finding the pulse had come down to 108, I left her asleep. The solution was directed without the antimonial. 10th. My father called at the house at six, A. M.; but learning that she was asleep, he did not see her. Ten, A. M. She had frequent evacuations, and remained free from exacerbations of pain; but the soreness of the abdomen was still considerable; and the pulse, which was at 106, being full, it was judged most prudent to repeat the venæsection, and eleven ounces of blood were again drawn. *Evening.* The pain had not returned, and the tenderness of the abdomen had gradually decreased; Pulse 100; bowels open. 11th. She had passed a good night, the soreness of the abdomen had quite gone off, and the pulse was at 98. The secretion of milk, and the discharge of lochia, both of which had been small before the attack of the disease, had returned afresh, and leave was given to have the breasts gently drawn. There was now every reason to hope that the disease was subdued, and that nothing remained to be done, but to keep the bowels in an open state, and to avoid the occasions of any fresh irritation. We were therefore not a little disappointed, to be informed that about four, P. M. the pain had returned with much severity. On inquiry, this relapse appeared to have been occasioned by too much exertion in suckling the child. The patient had fatigued herself by sitting up some time in bed, attempting to give the breast, and the pain immediately succeeded. The pulse had got up to 118. Fifteen ounces of blood were again taken from the arm; and no stool having been procured for five hours, the solution was ordered to be repeated every two hours. Ten P. M. The pains had been entirely removed since the bleeding; but the soreness still continuing, we were desirous of applying leeches to the abdomen. The lateness of the hour, however, made me prefer the application of cupping glasses; but I found the laxity of the integuments so great an impediment to this operation, that little blood was obtained. A warm fomentation was applied to the abdomen, which was very comfortable to the patient, and disposed her to sleep. The pulse was at 126. One evacuation only had been procured, and the solution was ordered to be continued. I remained with her till one o'clock, and then left her asleep. 12th. Visited early. The stools had been frequent; had disturbed her rest; and she seemed rather exhausted. Pulse 110. Nine A. M. She had slept an hour and a half, and was much refreshed. Pulse 100. The soreness had abated. The solution was directed to be taken in such quantity, as might procure a stool once in four hours. In the afternoon, the soreness not having gone off so completely as might be wished, ten leeches were applied to the abdomen, which afforded great relief. From this time to the 15th, nothing particular occurred. The solution was given as occasion required, and a saline draught in the intervals, both of which the patient always found refreshing to her; and she continued gradually recovering. On the 15th, at noon, there was a return of slight pains in the abdomen, accompanied with increased heat in the skin and a quick pulse. At two, P. M. I found the pulse nearly at 130; but the pain had gone off, and no soreness of the abdomen remained. The frequency of the pulse subsided as the heat declined; and in half an hour it had come down to 110. I did not apprehend any serious consequence from this slight attack; but at the request of the patient, eight leeches were applied to the abdomen. Her recovery was afterwards regularly progressive; but she was some time in regaining her strength. The progress of amendment was retarded by an attempt to regain her milk and suckle her child; but after a trial of some weeks, the attempt was then relinquished in consequence of its debilitating effect on the system.

We have here another instance of the necessity for *very copious* bleeding in some cases of puerperal fever. Thirty ounces of blood were taken away within four hours of the commencement of the disease; and a purging was early excited, which was maintained through its whole course; yet it did not appear to be subdued until fifty-one ounces of blood had been lost. On account of the return of pain, fifteen ounces more were taken; besides a small quantity by cupping, and the application of leeches; making in the whole, nearly seventy ounces. Should any one doubt whether the cure might not have been effected by a smaller loss of blood, at least it must be allowed, that large bleeding was proved to be safe; and it will scarcely be denied, that the inconvenience of a temporary debility is not to be put in competition with risquing the loss of life."

Under the head of general remarks, Mr. Hey enters into the consideration of those distinctions which authors have made between puerperal fever, peritonitis, and hysteritis. Also whether there is any difference, except in degree, between sporadic and epidemic puerperal fever. The result of his enquiries and observations may be gleaned from the following passage.

"But all its varieties, so far as I can judge from my experience and reading, may be reduced to two denominations, the sporadic, and the epidemic puerperal fever; in which I include inflammation of the uterus and peritoneum.

However Nosologists may think proper to describe the disease according to its seat &c.; I am persuaded that no other distinction is of any real practical importance. Nor is *this* of any further consequence, than that the epidemic disease requires more prompt attention, and more vigorous treatment. The means of cure are precisely the same in both." 181.

The perusal of Mr. Hey's volume is well calculated to afford the most important instruction. It will lop another head from the hydra DEBILITY. It will open the eyes of some of those Bœotian routinists who prescribe for a name, and *legally murder* their unhappy patients!

---

